# Observation of tunable accidental bound state in the continuum in silicon nanodisk array

**DOI:** 10.1515/nanoph-2023-0891

**Published:** 2024-03-06

**Authors:** Yingying Han, Lei Xiong, Jianping Shi, Guangyuan Li

**Affiliations:** College of Physics and Electronic Information, 12514 Anhui Normal University, Wuhu 241000, China; CAS Key Laboratory of Human-Machine Intelligence-Synergy Systems, 85411 Shenzhen Institute of Advanced Technology, Chinese Academy of Sciences, Shenzhen 518055, China; Shenzhen College of Advanced Technology, University of Chinese Academy of Sciences, Shenzhen, 518055, China

**Keywords:** accidental bound state in the continuum, all-dielectric metasurface, multipolar interference

## Abstract

We experimentally demonstrate the tuning of accidental bound states in the continuum (A-BICs) in silicon nanodisk arrays. The A-BIC emerges of the destructive interference of multipoles, which are the dominating out-of-plane electric dipole and in-plane magnetic dipole, and weak electric quadrupole and magnetic quadrupole. We further show that the spectral and angular position of the A-BIC can be conveniently tuned by varying the nanodisk size or the lattice period. Remarkably, the angular position can be tuned even to 0°, suggesting an interesting transition of the A-BIC from an off-Γ-BIC to an at-Γ-BIC. Our work provides a new strategy for light trapping with high quality factors, and the obtained tunable A-BICs can find potential applications in low-threshold lasing, enhanced nonlinear optics, and optical sensing.

## 1 Introduction

Bound states in the continuum (BICs) represent localized states with energies embedded in the continuous spectrum of radiating waves [1]. By adopting BICs, the physics and applications of all-dielectric resonant nanophotonics could be extended substantially [2], [3], [4]. As a simple way to achieve very large quality factors (*Q* factors) and to enhance light–matter interactions, BICs have found promising applications in low-threshold lasing [5], [6], [7], enhanced nonlinear optics [8], [9], [10], [11], strong chiroptical responses [12], [13], [14], ultrasensitive sensing [15], [16], [17], and active photonics [18], [19].

BICs can be classified into symmetry-protected BICs (SP-BICs) and accidental BICs (A-BICs) depending on the mechanisms of preventing the radiation [1], [2]. For the SP-BIC, the coupling to the radiation is forbidden when the system exhibits a reflection or rotational symmetry. Therefore, it is convenient to realize SP-BICs at the Γ point, which transfer into quasi-BICs if the structural symmetry is broken under normal incidence, or if the excitation field symmetry is broken with oblique incidence. In contrast, for the A-BIC, the coupling to the radiation is prevented by the destructive interference of coupled resonances at a specific set of system parameters, which is usually achieved via continuous parameter tuning. As a consequence, the SP-BICs have been widely investigated, whereas the A-BICs have received much less attention.

To date, efforts for the A-BICs supported by photonic crystals or metasurfaces, which are usually considered analogous, have been mainly put on the demonstration or the understanding of the formation mechanisms [20], [21], [22], [23], [24], [25], [26], [27], [28], [29], [30], [31], [32], [33], [34] and on the potential applications [35], [36], [37], [38]. This is probably because the conditions for the formation of A-BICs are not evident. In order to address this problem, Abujetas et al. recently established a general guideline for the formation of the A-BICs in metasurfaces, and numerically showed that the spectral and angular position of A-BICs can be tuned by the aspect ratio of the nanodisk [39]. However, the too narrow linewidths or too small modulation depths of the quasi-A-BICs hinder experimental demonstrations. In other words, the experimental demonstration of tuning the A-BICs, especially tuning it from an off-Γ-BIC to an at-Γ-BIC, has yet to be reported.

**Figure 1:**
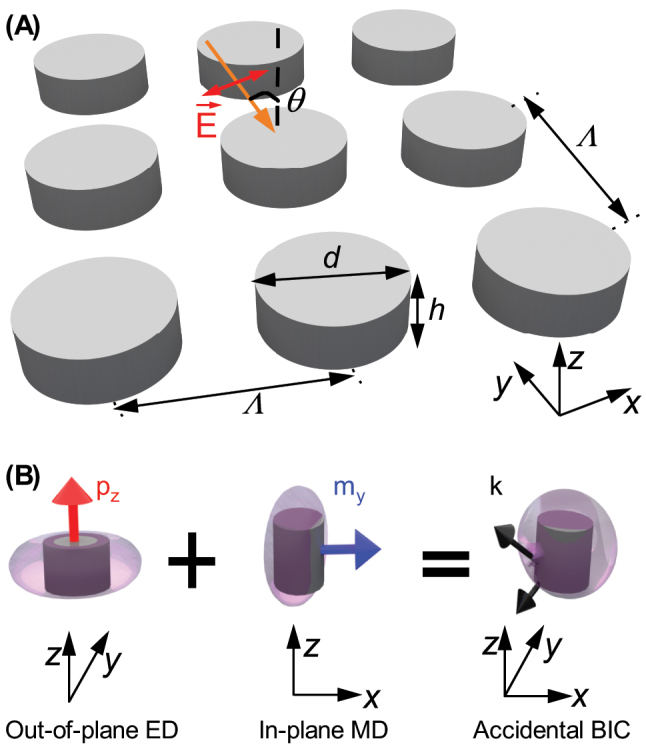
Schematics of the mechanism for realizing A-BIC emerged from destructive interference of multipoles in silicon metasurface. (A) Periodic silicon nanodisks with diameter *d*, height *h*, and periodicity Λ are illuminated by plane wave of incidence angle *θ* and electric field polarized in *x*–*z* plane. (B) Destructive interference between the dominant contributing ED in the *y*–*z* plane and MD in the *x*–*z* plane approximately results in the A-BIC at specific angle.

Here, we experimentally demonstrate the tuning of A-BICs in periodic silicon nanodisk array, as illustrated in Figure 1(A). We will show that the A-BIC emerges when the radiations of multipoles interference destructively and cancel at a specific position of wavelength and radiation angle, (*λ*_0_, *θ*_0_), as illustrated in Figure 1(B). We will, both numerically and experimentally, show that the position in (*ω*, *k*_‖_) space, or equivalently the position in terms of (*λ*_0_, *θ*_0_), can be conveniently tuned not only by varying the nanodisk’s diameter or height, consistent with [39], but also by changing the lattice period. More strikingly, we will show that *θ*_0_ can be tuned even to 0°, suggesting an interesting and new phenomenon of the A-BIC transition from an off-Γ BIC to an at-Γ-BIC.

## 2 Results and discussion

### 2.1 Spectral and near-field characteristics

Figure 1(A) illustrates the periodic array of silicon nanodisks with diameter *d*, height *h* = 200 nm, and square periodicity Λ. The nanodisk array embedded in homogeneous dielectric environment with refractive index *n*_0_ = 1.45 is illuminated by plane wave with incidence angle *θ* and electric field polarized in the *x*–*z* plane and of unitary amplitude (|*E*_0_| = 1). Note that in this work, *θ* means the incidence angle in free space.

We adopted a home-built package based on rigorous coupled-wave analysis developed following [40], [41], [42] to simulate the zeroth-order transmittance spectra and the near-field distributions of the periodic silicon nanodisks. In all the simulations, the wavelength-dependent refractive indices of silicon were taken from Palik [43].

**Figure 2:**
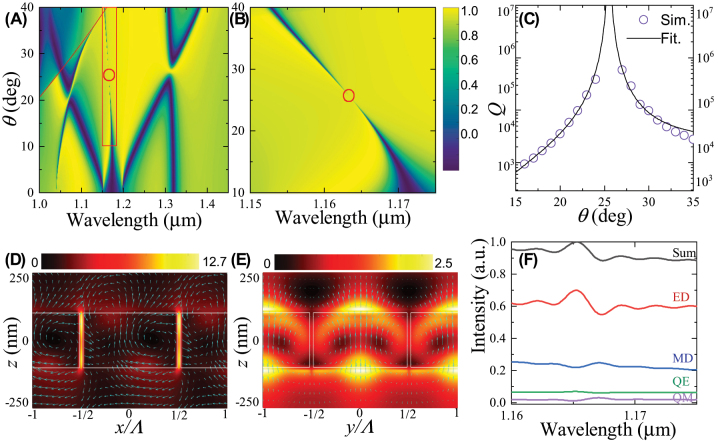
Spectral, near-field and far-field characteristics of the A-BIC. (A) Simulated angular-resolved transmittance spectra of the silicon nanodisk array. (B) Zoom of the red box in (A). Red circles in (A) and (B) indicate the occurrence of the A-BIC at the position of (*λ*_0_ = 1.164 μm, *θ*_0_ = 25°). (C) Extracted quality factors (open circles) of the resonance shown in (B) as functions of *θ*. Solid curves denote theoretical fitting with inverse square functions. (D) and (E) Near-field electric filed distributions |*E*/*E*_0_|^2^ (color for intensity and arrows for directions) for the A-BIC at (*λ*_0_, *θ*_0_), in (D) the *x*–*z* plane with *y* = 0 for the central panel, and (E) in the *y*–*z* plane with *x* = 0 for the bottom panel. Silicon nanodisks are outlined by white boxes. (F) Multipolar contents of the electromagnetic fields at the A-BIC. “ED,” “MD,” “EQ,” “MQ,” and “Sum” stand for electric dipole, magnetic dipole, electric quadrupole, magnetic quadrupole, and sum of all these multipoles, respectively.

Figure 2(A) depicts the simulated angular-resolved transmittance spectra of the silicon nanodisk array with Λ = 555 nm and *d* = 535 nm. Strictly speaking, the structure under study is in the photonic crystal phase rather than in the metasurface phase based on the physics of Mie and Bragg resonances [44] (Figures S1 in the Supplementary). Results show that for *θ* = 10°, there exist five transmittance dips within the spectral range between 1.0 μm and 1.45 μm. When the first two dips or the last two dips are spectrally overlapped around *θ* = 20° or *θ* = 26°, respectively, nearly unitary transmittance and thus nearly zero reflectance can be obtained. This suggests multiple resonant lattice Kerker effects, which can be treated as the extension of our previous work [45]. This phenomenon is out of the scope of this work and will be reported elsewhere.

Interestingly, we find that as *θ* increases, the third dip first gradually narrows down, disappears around *θ*_0_ = 25°, as indicated by the open circle, and emerges again with gradually increasing linewidth. This is better visualized by the zoomed view in Figure 2(B). We further extracted the *Q* factors and plot them as functions of the incidence angle in Figure 2(C). Results show that as *θ* approaches 25°, the *Q* factor increases significantly and tends to infinity. Indeed, the dependence of these *Q* factors on *θ* is inverse quadratic, as shown by the good agreement between the simulated values and the fitting curves, which follow

(1) $$Q\propto 1/\theta^{2}.$$

Therefore, the spectral behaviors, as well as the inverse quadratic relationships, suggest the occurrence of an A-BIC at *θ*_0_ = 25°, and the disappearance of the transmittance dip with infinite *Q* factor suggests that, the A-BIC is an “ideal” BIC in theory [46].

In order to understand the origin of this A-BIC with clear physical intuition, we turn to the near-field optical pictures and the multipolar decomposition. We emphasize here that the physical understanding of A-BIC based on the multipolar decomposition of electromagnetic fields is acceptable not only in metasurfaces but also in photonic crystals [7]. Figure 2(D) shows that, for the A-BIC at (*λ*_0_ = 1.164 μm, *θ*_0_ = 25°), the electric field in the *x*–*z* plane is mainly confined to the gaps between the silicon nanodisks, and its circulation within a unit cell forms an in-plane magnetic dipole (MD). On the other hand, Figure 2(E) shows that the electric field in the *y*–*z* plane is mainly confined to the central outside of the silicon nanodisks, forming an evident out-of-plane electric dipole (ED). The multipolar decomposition in Figure 2(F) verifies that dominant contributing contents are the ED and the MD, accompanied with relatively weak electric quadrupole (EQ) and magnetic quadrupole (MQ), which slightly complex the near-field distributions in Figure 2(D) and (E). Therefore, both the near-field distributions and the multipolar decomposition have revealed that the formation of the A-BIC can be understood by the approximately destructive interference of the dominating out-of-plane electric dipole and in-plane magnetic dipole, and weak electric and magnetic quadrupoles, which is similar to the literature [7].

As the incidence angle varies, the relative strengths of the multipolar contents vary (Figure S2 in the Supplementary). Nevertheless, the dominant contributing multipoles remain the out-of-plane ED and the in-plane MD, as illustrated by Figure 1(B). Therefore, the spectral and angular position of the A-BIC, (*λ*_0_, *θ*_0_), can be approximately determined by solving the following conditions expressed as [39]

(2) $$Re\left[\frac{1}{k^{2}\alpha_{y}^{\left(m\right)}}\right]=Re\left[G_{\mathrm{byy}}+\frac{k}{k_{x}}G_{\mathrm{byz}}\right],$$

(3) $$Re\left[\frac{1}{k^{2}\alpha_{z}^{\left(e\right)}}\right]=Re\left[G_{\mathrm{bzz}}+\frac{k_{x}}{k}G_{\mathrm{byz}}\right],$$

which should be satisfied simultaneously. Here, *G*_*bij*_ are the matrix elements of the lattice depolarization Green dyadic ${\overset{↔}{G}}_{b}$ , and $\alpha_{y}^{\left(m\right)}$ and $\alpha_{z}^{\left(e\right)}$ are the polarizabilities of the MD along the *y* axis and the ED along the *z* axis.

### 2.2 Experimental validations

**Figure 3:**
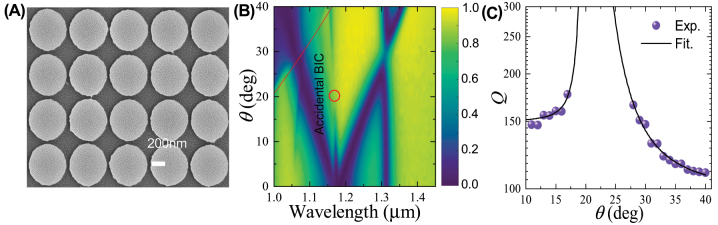
Experimental demonstration of the A-BIC. (A) SEM image and (B) measured transmittance spectra of as-fabricated silicon nanodisk array with Λ = 555 nm and *d* = 535 nm. (C) Measured *Q* factors of quasi-A-BICs, i.e., the resonances corresponding to the nearly vertical transmittance dips around the red circle, as functions of *θ*. Solid curve denotes theoretical fitting with inverse square functions.

To verify our numerical findings, we fabricated periodic silicon nanodisks on a quartz substrate using the state-of-art nanofabrication techniques, which involve the electron-beam lithography and deposition, liftoff, dry etching, and wet etching processes. The as-fabricated samples, of which a typical scanning electron image is shown in Figure 3(A), were then covered with index-matching oil in order to guarantee homogeneous dielectric environment surrounding the silicon nanodisks. The detailed descriptions on the fabrication and the optical characterization can be found in our previous work [47].

Figure 3(B) plots the measured angular-resolved transmittance spectra for *θ* varying from 0° to 40°. Comparing Figure 2(A) and (B), we find that in general, the measured transmittance spectra agree well with the simulation results. Remarkably, as *θ* increases from 0° to 40°, the nearly vertical transmittance dip first narrows down, disappears around *θ*_0_ = 20°, which is slightly smaller than the theoretical position (*θ*_0_ = 25° in Figure 2(A)), and emerges again with increasing linewidth. This suggests the occurrence of the A-BIC, as indicated by the red circle.

We further extracted the quality factors of the quasi-A-BIC around *θ*_0_ = 20° from the measured transmittance spectra in Figure 3(B). Figure 3(C) shows that the quality factor tends to increase significantly as *θ* approaches 20°. When *θ* becomes too close to 20°, the transmittance dip has so small modulation depth that it is difficult to extract the quality factor from the transmittance spectra. The dependence of the measured quality factor on the incidence angle also follows the inverse quadratic relationships that can be expressed in Equation (1). These behaviors are similar to the simulation results shown in Figure 2(C) except that the measured quality factors are smaller than the simulated results. The degraded quality factors in experiments may attribute to the finite size of samples, material absorption, surface scattering [46], and fabrication imperfections, which include variations in the nanodisks’ sizes and shapes as shown by Figure 3(A), and side-wall tilting, as well as the use of incoherent source in measurements, which greatly reduces the measurable *Q* factors, as demonstrated by Bin-Alam et al. [48]. Therefore, we have experimentally validated the occurrence of the A-BIC in the periodic silicon nanodisks and shown that it is not an “ideal” BIC in practice.

### 2.3 Tuning A-BIC via nanodisk size or lattice period

**Figure 4:**
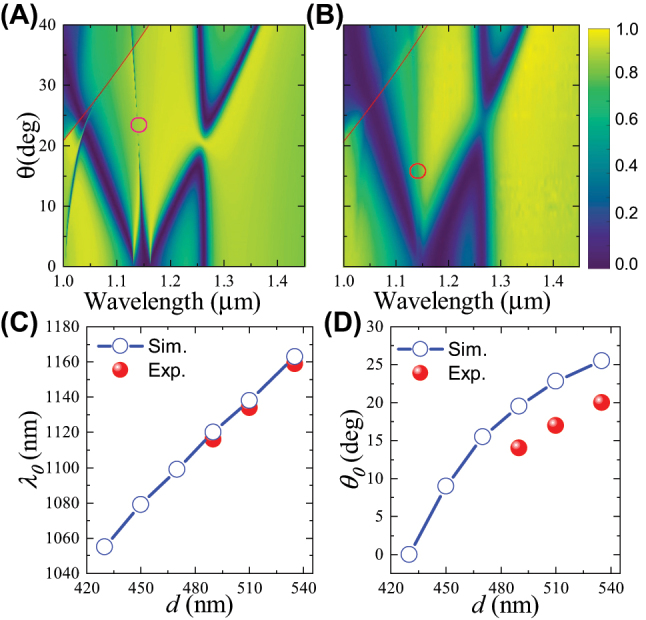
Tuning the A-BIC via the nanodisk diameter. (A) Simulated and (B) measured angular-resolved transmittance spectra of silicon nanodisk array with Λ = 555 nm and *d* = 510 nm. (C) and (D) Comparison of simulated (circles) and measured (balls) results on the (C) spectral and (D) angular positions for the A-BICs as functions of the nanodisk diameter *d*.

By decreasing the nanodisk diameter to *d* = 510 nm while keeping the lattice period to be Λ = 555 nm, we plot the simulated and measured angular-resolved transmittance spectra in Figure 4(A) and (B), respectively. Results show that for the third resonance, the A-BIC occurs at *θ*_0_ = 22° and *λ*_0_ = 1.138 μm in simulations, or *θ*_0_ = 17° and *λ*_0_ = 1.134 μm in experiments. Comparing with the results for *d* = 535 nm in Figure 2(A) and (B), we find that the spectral and angular position (*λ*_0_, *θ*_0_) for the A-BIC is shifted to the left bottom for both the simulation and the experimental results.

We summarize the extracted spectral and angular positions for the A-BICs from the simulated and measured angular-resolved transmittance spectra for different nanodisk diameters (Figures S3 and S4 in the Supplementary) and plot the results in Figure 4(C) and (D). Results show that the spectral and angular position (*λ*_0_, *θ*_0_) for the A-BIC keeps increasing with the nanodisk diameter, consistent with the results in [39]. This behavior is experimentally demonstrated by the good agreement between the simulated and measured positions of (*λ*_0_, *θ*_0_). Strikingly, when the diameter decreases to *d* = 430 nm, *θ*_0_ can even reduce to 0°. This suggests an interesting transition of the A-BIC from the off-Γ-BIC to the at-Γ-BIC, which is a new phenomenon that has not been reported yet.

Similarly, the spectral and angular position (*λ*_0_, *θ*_0_) for the A-BIC also decreases with the nanodisk’s height (Figure S5 in the Supplementary). This behavior is also consistent with the results in [39].

**Figure 5:**
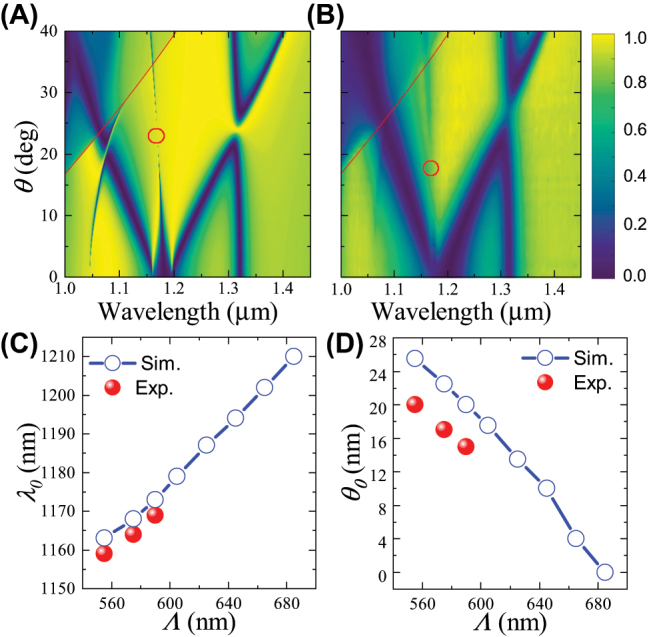
Tuning the A-BIC via the lattice period. (A) Simulated and (B) measured angular-resolved transmittance spectra of silicon nanodisk array with Λ = 575 nm and *d* = 535 nm. (C) and (D) Comparison of simulated (circles) and measured (balls) results on the (C) spectral and (D) angular positions for the A-BICs as functions of the lattice period Λ.

Besides the nanodisk diameter and height, we find that (*λ*_0_, *θ*_0_) for the A-BIC can also be tuned by varying the lattice period, which was not shown in [39]. Figure 5(A) shows that if the lattice period increases to Λ = 575 nm while keeping the nanodisk diameter to be *d* = 535 nm, the angular position for the A-BIC decreases to *θ*_0_ = 22°, whereas the spectral position increases to *λ*_0_ = 1.168 μm. The simulated angular-resolved transmittance spectra are also validated by the experimental results, as shown by Figure 5(B), where good agreement can be found except that the measured position for the A-BIC (*λ*_0_, *θ*_0_) is also slightly smaller than the simulated data.

By further increasing Λ, we find that *λ*_0_ keeps increasing, whereas *θ*_0_ keeps reducing, as shown by Figures 5(C) and (D) and S6 and S7 in the Supplementary. Similarly to Figure 4(D), we also find that *θ*_0_ can even reduce to 0° when Λ = 685 nm. In other words, the transition of the A-BIC from the off-Γ-BIC to the at-Γ-BIC can also be observed by increasing the lattice period.

Although the above behaviors of the spectral and angular position for the A-BIC as the nanodisk’s size or the lattice period varies can be approximately predicted by the theoretical expressions of Equations (2) and (3) that are developed in [39], here we turn to qualitative explanations with more straightforward physical pictures. For smaller nanodisk’s diameter or height or larger lattice period, the delocalized nature of the multipoles becomes more important. Therefore, if the nanodisk’s diameter or height decreases, the resonance wavelength is blue shifted, resulting in the blue-shifted A-BIC. On the other hand, the more contribution from the delocalization leads to decreasing *θ*_0_ for the A-BIC, that is *θ*_0_ being closer to 0°. If the lattice period increases, the resonance is red-shifted, resulting in red-shifted A-BIC. On the other hand, the more contribution from the delocalization also leads to decreasing *θ*_0_ for the A-BIC. In other words, with the characteristics of delocalization, we explained the tuning of the spectral and angular position for the A-BIC, as well as the transition of the A-BIC from the off-Γ-BIC to the at-Γ-BIC through varying the nanodisk’s size or the lattice period.

## 3 Conclusions

In conclusions, we have experimentally demonstrated the tuning of the spectral and angular position of the A-BICs in periodic silicon nanodisks by varying the geometric size, which is the nanodisk’s diameter or height, or the lattice period. We have validated these results with good agreement between simulation results and experimental data. Strikingly, we have further numerically shown that the angular position of the A-BIC can be tuned even to 0°, suggesting an interesting transition of the A-BIC from an off-Γ-BIC to an at-Γ-BIC. We expect that this work will advance the understanding of the A-BIC and promote its applications.

## Supplementary Material

Supplementary Material Details

## References

[1] Hsu C. W., Zhen B., Stone A. D., Joannopoulos J. D., Soljačić M. (2016). Bound states in the continuum. *Nat. Rev. Mater.*.

[2] Koshelev K., Bogdanov A., Kivshar Y. (2019). Meta-optics and bound states in the continuum. *Sci. Bull.*.

[3] Azzam S. I., Kildishev A. V. (2021). Photonic bound states in the continuum: from basics to applications. *Adv. Opt. Mater.*.

[4] Joseph S., Pandey S., Sarkar S., Joseph J. (2021). Bound states in the continuum in resonant nanostructures: an overview of engineered materials for tailored applications. *Nanophotonics*.

[5] Kodigala A., Lepetit T., Gu Q., Bahari B., Fainman Y., Kanté B. (2017). Lasing action from photonic bound states in continuum. *Nature*.

[6] Ha S. T. (2018). Directional lasing in resonant semiconductor nanoantenna arrays. *Nat. Nanotechnol.*.

[7] Hwang M.-S. (2021). Ultralow-threshold laser using super-bound states in the continuum. *Nat. Commun.*.

[8] Carletti L., Koshelev K., De Angelis C., Kivshar Y. (2018). Giant nonlinear response at the nanoscale driven by bound states in the continuum. *Phys. Rev. Lett.*.

[9] Liu Z. (2019). High-*Q* quasibound states in the continuum for nonlinear metasurfaces. *Phys. Rev. Lett.*.

[10] Koshelev K., Tang Y., Li K., Choi D.-Y., Li G., Kivshar Y. (2019). Nonlinear metasurfaces governed by bound states in the continuum. *ACS Photonics*.

[11] Liu Z., Wang J., Chen B., Wei Y., Liu W., Liu J. (2021). Giant enhancement of continuous wave second harmonic generation from few-layer GaSe coupled to high-*Q* quasi bound states in the continuum. *Nano Lett.*.

[12] Gorkunov M. V., Antonov A. A., Kivshar Y. S. (2020). Metasurfaces with maximum chirality empowered by bound states in the continuum. *Phys. Rev. Lett.*.

[13] Kim K.-H., Kim J.-R. (2021). High-*Q* chiroptical resonances by quasi-bound states in the continuum in dielectric metasurfaces with simultaneously broken in-plane inversion and mirror symmetries. *Adv. Opt. Mater.*.

[14] Shi T. (2022). Planar chiral metasurfaces with maximal and tunable chiroptical response driven by bound states in the continuum. *Nat. Commun.*.

[15] Romano S. (2018). Label-free sensing of ultralow-weight molecules with all-dielectric metasurfaces supporting bound states in the continuum. *Photonics Res.*.

[16] Yesilkoy F. (2019). Ultrasensitive hyperspectral imaging and biodetection enabled by dielectric metasurfaces. *Nat. Photonics*.

[17] Romano S. (2020). Ultrasensitive surface refractive index imaging based on quasi-bound states in the continuum. *ACS Nano*.

[18] Han S. (2019). All-Dielectric active terahertz photonics driven by bound states in the continuum. *Adv. Mater.*.

[19] Abujetas D. R., de Sousa N., García-Martín A., Llorens J. M., Sánchez-Gil J. A. (2021). Active angular tuning and switching of Brewster quasi bound states in the continuum in magneto-optic metasurfaces. *Nanophotonics*.

[20] Marinica D. C., Borisov A. G., Shabanov S. V. (2008). Bound states in the continuum in photonics. *Phys. Rev. Lett.*.

[21] Hsu C. W., Zhen B., Chua S.-L., Johnson S. G., Joannopoulos J. D., Soljačić M. (2013). Bloch surface eigenstates within the radiation continuum. *Light: Sci. Appl.*.

[22] Hsu C. W. (2013). Observation of trapped light within the radiation continuum. *Nature*.

[23] Yang Y., Peng C., Liang Y., Li Z., Noda S. (2014). Analytical perspective for bound states in the continuum in photonic crystal slabs. *Phys. Rev. Lett.*.

[24] Ni L., Wang Z., Peng C., Li Z. (2016). Tunable optical bound states in the continuum beyond in-plane symmetry protection. *Phys. Rev. B*.

[25] Neale S., Muljarov E. A. (2021). Accidental and symmetry-protected bound states in the continuum in a photonic-crystal slab: a resonant-state expansion study. *Phys. Rev. B*.

[26] Bulgakov E. N., Sadreev A. F. (2014). Bloch bound states in the radiation continuum in a periodic array of dielectric rods. *Phys. Rev. A*.

[27] Lepetit T., Kanté B. (2014). Controlling multipolar radiation with symmetries for electromagnetic bound states in the continuum. *Phys. Rev. B*.

[28] Bulgakov E. N., Maksimov D. N. (2017). Topological bound states in the continuum in arrays of dielectric spheres. *Phys. Rev. Lett.*.

[29] Sadrieva Z., Frizyuk K., Petrov M., Kivshar Y., Bogdanov A. (2019). Multipolar origin of bound states in the continuum. *Phys. Rev. B*.

[30] Gladyshev S., Shalev A., Frizyuk K., Ladutenko K., Bogdanov A. (2022). Bound states in the continuum in multipolar lattices. *Phys. Rev. B*.

[31] Overvig A. C., Malek S. C., Carter M. J., Shrestha S., Yu N. (2020). Selection rules for quasi-bound states in the continuum. *Phys. Rev. B*.

[32] Han S., Pitchappa P., Wang W., Srivastava Y. K., Rybin M. V., Singh R. (2021). Extended bound states in the continuum with symmetry-broken terahertz dielectric metasurfaces. *Adv. Opt. Mater.*.

[33] Sidorenko M. S. (2021). Observation of an accidental bound state in the continuum in a chain of dielectric disks. *Phys. Rev. Appl.*.

[34] Zhou C. (2023). Bound states in the continuum in asymmetric dielectric metasurfaces. *Laser Photonics Rev.*.

[35] Jin J., Yin X., Ni L., Soljačić M., Zhen B., Peng C. (2019). Topologically enabled ultrahigh-*Q* guided resonances robust to out-of-plane scattering. *Nature*.

[36] Kang M., Mao L., Zhang S., Xiao M., Xu H., Chan C. T. (2022). Merging bound states in the continuum by harnessing higher-order topological charges. *Light: Sci. Appl.*.

[37] Yang C., Sang T., Li S., Wang Y., Cao G., Hu L. (2022). Tailoring the light absorption of monolayer graphene via accidental quasi-bound states in the continuum. *J. Opt. Soc. Am. B*.

[38] Zhang Y., Liu X., Zhao R., Li J. (2023). Unidirectional asymmetry transmission based on quasi-accidental bound states in the continuum. *Phys. Chem. Chem. Phys.*.

[39] Abujetas D. R., Olmos-Trigo J., Sánchez-Gil J. A. (2022). Tailoring accidental double bound states in the continuum in all-dielectric metasurfaces. *Adv. Opt. Mater.*.

[40] Moharam M. G., Pommet D. A., Grann E. B., Gaylord T. K. (1995). Stable implementation of the rigorous coupled-wave analysis for surface-relief gratings: enhanced transmittance matrix approach. *J. Opt. Soc. Am. A*.

[41] Lalanne P. (1997). Improved formulation of the coupled-wave method for two-dimensional gratings. *J. Opt. Soc. Am. A*.

[42] David A., Benisty H., Weisbuch C. (2006). Fast factorization rule and plane-wave expansion method for two-dimensional photonic crystals with arbitrary hole-shape. *Phys. Rev. B*.

[43] Palik E. D. (1988). *Handbook of Optical Constants of Solids*.

[44] Rybin M. V., Filonov D. S., Samusev K. B., Belov P. A., Kivshar Y. S., Limonov M. F. (2015). Phase diagram for the transition from photonic crystals to dielectric metamaterials. *Nat. Commun.*.

[45] Xiong L. (2023). Polarization-controlled dual resonant lattice Kerker effects. *Nano Res.*.

[46] Sadrieva Z. F. (2017). Transition from optical bound states in the continuum to leaky resonances: role of substrate and roughness. *ACS Photonics*.

[47] Du X., Xiong L., Zhao X., Chen S., Shi J., Li G. (2022). Dual-band bound states in the continuum based on hybridization of surface lattice resonances. *Nanophotonics*.

[48] Bin-Alam M. S. (2021). Ultra-high-*Q* resonances in plasmonic metasurfaces. *Nat. Commun.*.

