# Supplementary material for: Observation of tunable accidental bound state in the continuum in silicon nanodisk array
Source: Nanophotonics. 2024 Mar 6;13(9):1603–9. doi: 10.1515/nanoph-2023-0891 (PMC11635966; doi:10.1515/nanoph-2023-0891)
Supplement: Supplementary file 1 — Supplementary Material Details [file j_nanoph-2023-0891_suppl_001.pdf]

Supplementary Material

Observation of tunable accidental bound state in the continuum in silicon nanodisk array

Yingying Han, Lei Xiong, Jianping Shi*, and Guangyuan Li*

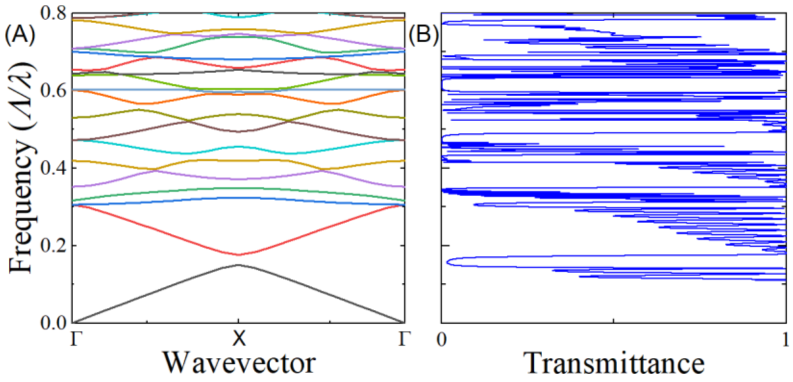

Figure S1. (A) Band structure and (B) transmittance spectra for the silicon nanodisk array with d = 535 nm and Λ = 555 nm for TM polarization.

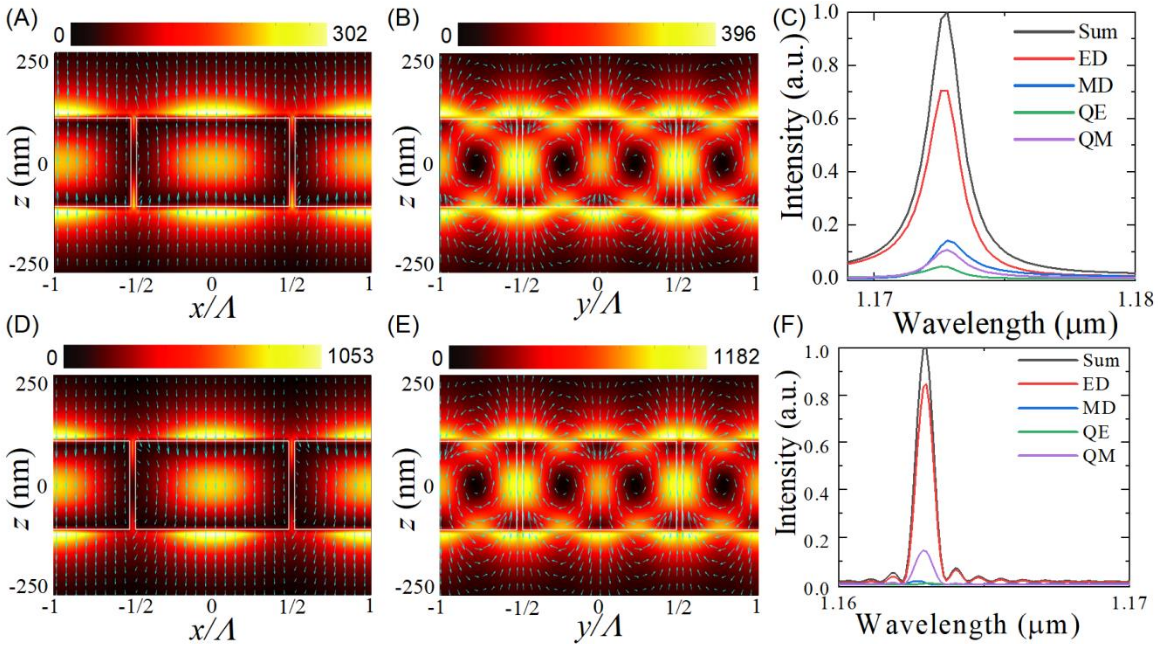

Figure S2. (A)(B)(D)(E) Near-field electric filed distributions $|E/E_0|^2$ (color for intensity and arrows for directions) for (A)(B) θ = 15° and (D)(E) θ = 30°, in (A)(D) the *x*-*z* plane with *y* = 0, and (B)(E) the *y*-*z* plane with *x* = 0. Silicon nanodisks are outlined by white boxes. (C)(F) Multipole decomposition for (C) θ = 15° and (F) θ = 30°.

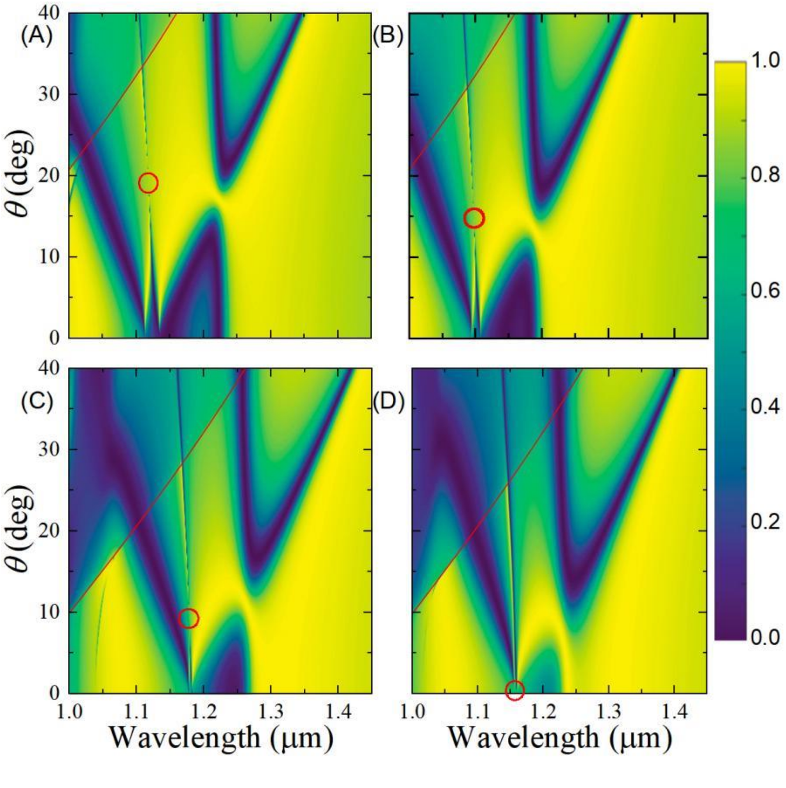

Figure S3. Simulated angular-resolved transmittance spectra of the silicon metasurface with Λ = 555 nm and (A) *d* = 490 nm, (B) *d* = 470 nm, (C) *d* = 450 nm, (D) *d* = 430 nm.

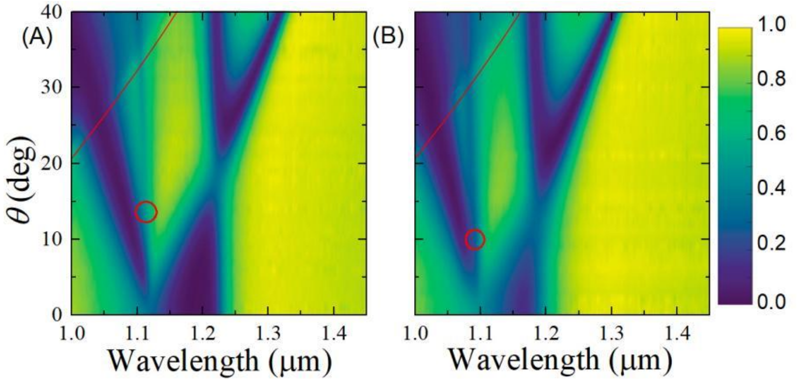

Figure S4. Measured angular-resolved transmittance spectra of the silicon metasurface with Λ = 555 nm and (A) d = 490 nm, (B) d = 470 nm.

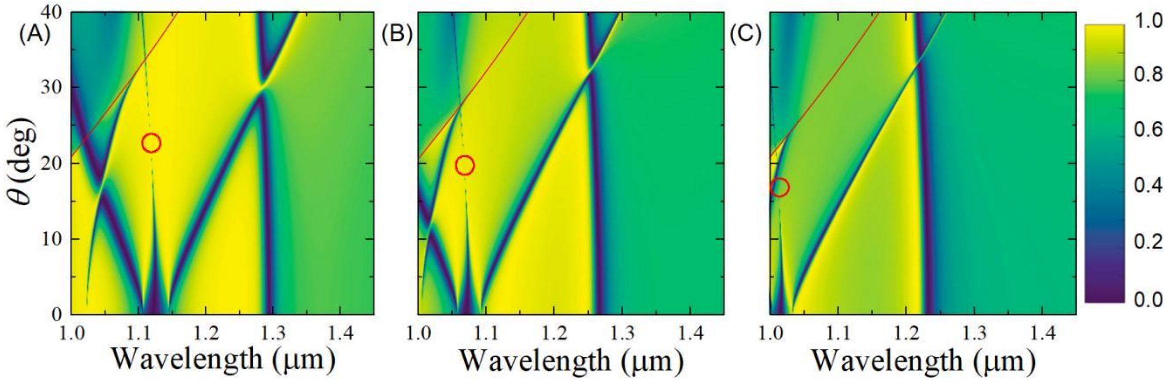

Figure S5. Simulated angular-resolved transmittance spectra of the silicon metasurface with Λ = 555 nm and d = 535 nm. (A) h = 180 nm, (B) h = 160 nm, (C) h = 140 nm.

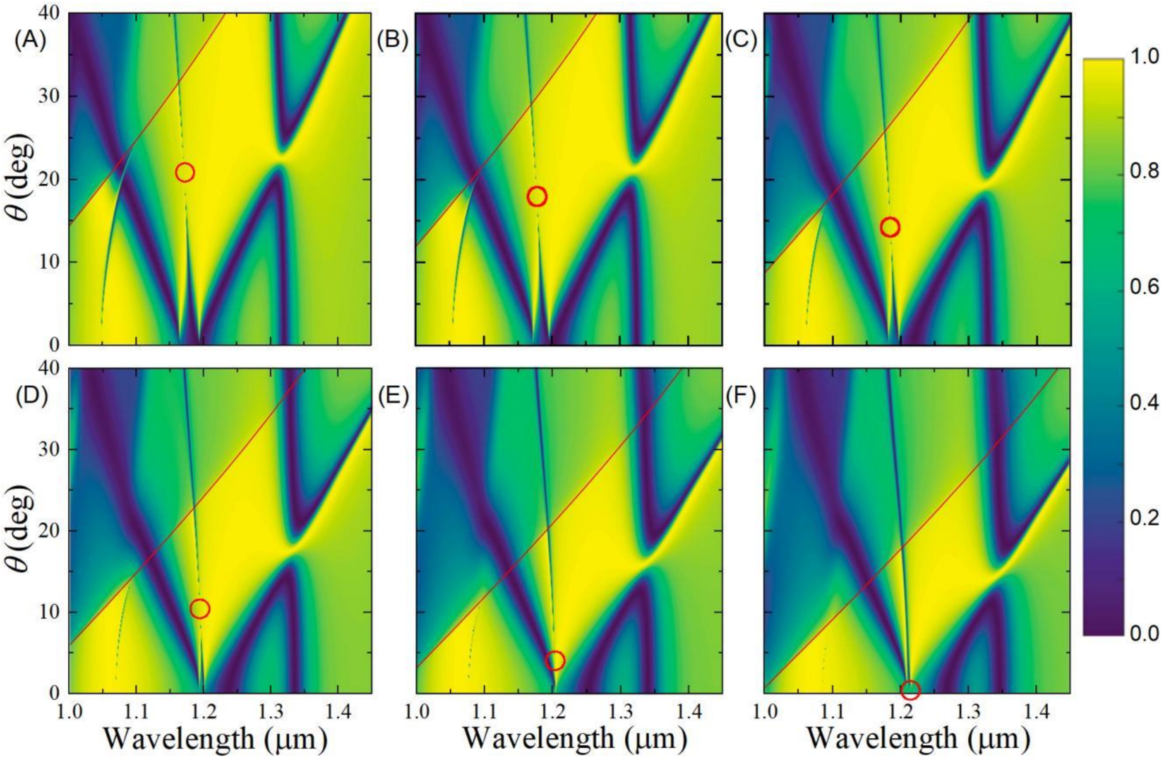

Figure S6. Simulated angular-resolved transmittance spectra of the silicon metasurface with d = 535 nm and (A) Λ = 590 nm, (B) Λ = 605 nm, (C) Λ = 625 nm, (D) Λ = 645 nm, (E) Λ = 665 nm, (F) Λ = 685 nm.

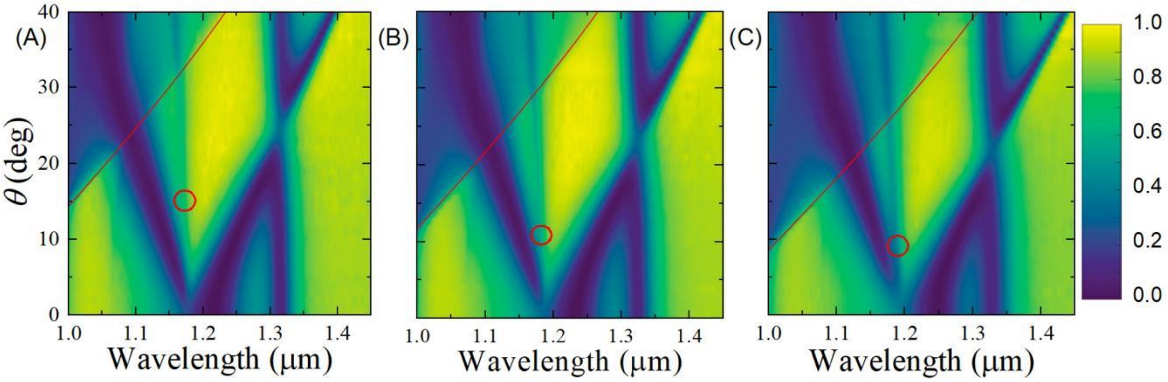

Figure S6. Measured angular-resolved transmittance spectra of the silicon metasurface with d = 535 nm, and (A) Λ = 590 nm, (B) Λ = 605 nm, (C) Λ = 625 nm.

Reference:

[1] Supplementary Material of X. Fang, L. Xiong, J. Shi, and G. Li. "High-Q quadrupolar plasmonic lattice resonances in horizontal metal-insulator-metal gratings". *Opt. Lett.* vol. 46, 2021, pp. 1546-1549.

[2] Supplementary Material of M. S. Bin-Alam, O. Reshef, Y. Mamchur, et al., "Ultra-high-Q resonances in plasmonic metasurfaces," *Nat. Commun.*, vol. 12, 2021, pp. 974.
